# Patterns of community follow-up, subsequent health service use and survival among young and mid-life adults discharged from chronic care hospitals: a retrospective cohort study

**DOI:** 10.1186/s12913-016-1631-z

**Published:** 2016-08-13

**Authors:** Kerry Kuluski, Sima Gandhi, Christina Diong, Carolyn Steele Gray, Susan E. Bronskill

**Affiliations:** 1 Lunenfeld-Tanenbaum Research Institute, Sinai Health System (Bridgepoint Hospital Site), 1 Bridgepoint Drive, Toronto, ON M4M 2B5 Canada; 2 Institute of Health Policy, Management and Evaluation, Dalla Lana School of Public Health, University of Toronto, 155 College Street, Suite 425, Toronto, ON M5T 3 M6 Canada; 3 Institute for Clinical Evaluative Sciences, G123, 2075 Bayview Avenue, Toronto, ON M4N 3 M5 Canada

## Abstract

**Background:**

Despite the demand for rehabilitation and chronic care services *across* the life course, policy and care strategies tend to focus on older adults and overlook medically complex younger adult populations. This study examined young and mid-life adults discharged from tertiary chronic care hospitals in order to describe their health service use and to examine the association between patterns of timely community follow-up, and subsequent health outcomes.

**Methods:**

This population-based retrospective cohort study used linked administrative data to identify 1,906 individuals aged 18–64 years and discharged alive from tertiary chronic care hospitals in Ontario, Canada between April 1, 2005 and March 31, 2006. Multivariate Cox proportional hazard models were used to examine the effect of community follow-up within 7 days of discharge (home care and/or a primary care physician visit or neither) on time to first hospitalization and emergency department (ED) visit. Five-year survival was examined using Kaplan-Meier survival curves.

**Results:**

The cohort had a high prevalence of multi-morbidity and use of hospital, emergency services and physician services was high in the year following discharge. Most individuals received follow-up care from a primary care physician and/or home care within 7 days of discharge while 30 % received neither. Within 1 year of discharge, 18 % of individuals died. Among those who survived, time to acute care hospitalization in the year following discharge was significantly longer among those who received both a home care and a physician follow-up visit compared to those who received neither. No significant associations were found between community follow-up and ED visits within 1 year.

**Conclusions:**

Immediate community follow-up may reduce subsequent use of acute care services. Future research should determine why some individuals, who would likely benefit from services, are not receiving them including barriers to access.

## Background

Chronic disease (i.e., long-term conditions) touches individuals at every age and impacts both physical [1, 2] and psycho-social functioning [3]. Despite the prevalence of chronic disease *across* the life course, policy and care strategies tend to focus on older adults; typically 65 years and over. For instance, integrated models of community based care as well as strategies to improve care transitions from hospital to home have typically been developed for older adults with specific diagnoses such as heart failure [4] or COPD [5] with some attention to older adults with multimorbidity [6, 7]. Less attention has been paid to the *under 65* population who may have similar clinical profiles as older adults despite being much younger. In this paper we focus on people who are young and mid-life adults with complex care needs. We define complex care needs as having two or more chronic health problems (i.e., or multimorbidity) such as multiple sclerosis, osteoporosis and heart disease simultaneously and requiring ongoing rehabilitation and supportive care as a result [8]. While there tends to be no dominant disease clusters within populations of people with multimorbidity [9] a recent study showed a clustering of specific physical and mental health conditions in the under 65 population [10].

It is important to focus on young and mid-life adults due to the unique challenges they may face relative to their older counterparts including the potential number of years that they will spend in the health care system, as well as the timing of their illness, which are occurring when social roles responsibilities related to employment and family may be at their peak [11–14].

Some care settings, such as tertiary chronic care hospitals, have a natural cluster of people with complex care needs. In Ontario, the term complex continuing care (CCC) is used to describe the tertiary care setting that delivers “medically complex and specialized services.” CCC beds are located in dedicated freestanding facilities or in designated beds within acute care hospitals [15]. While the term CCC is unique to Ontario, in other jurisdictions it is akin to tertiary chronic or intermediate level care. Unlike residential long-term care or skilled nursing facilities, many people in CCC are under 65 years of age -- one in six individuals who receive treatment in a CCC bed in Canada are between 19 and 64 years [15].

People are referred to CCC following an acute hospital stay [16]; for instance, if a person undergoes surgery for hip fracture but requires additional rehabilitation due to underlying illnesses and poor recovery time they would be referred to CCC. Compared to their older adult counterparts, young adults in CCC tend to be more clinically stable, totally dependent on others to perform activities of daily living, have longer lengths of stay, and more likely to be discharged to the community than residential long-term care [15]. While the intent of CCC is to restore (at least some) physical and/or cognitive function and stabilize health, when people are discharged from this setting it is assumed that some level of oversight and management of health issues is required.

Several studies have documented home care (including personal support, homemaking, occupational therapy and physical therapy) and physician follow-up as an important component of care continuity for individuals following a hospital stay. In particular, when indicated, these services have been shown to reduce the risk of repeat hospitalizations [17–21] and ED use [4]. Risk of death can also decrease with community follow-up [5], particularly if a similar care provider (i.e., physician) is involved in the care of the person in hospital and in the community [22]. The identified literature in this area, however, tends to focus on older adults, typically with a specific disease (e.g., heart failure) discharged from acute care settings. It is unclear if community follow-up impacts utilization and mortality for young and mid-life adults discharged from tertiary chronic care. This is an unfortunate oversight given the substantial and ongoing health care needs that are typically seen in this population.

Despite current health policies that emphasize the importance of linking individuals to care providers in the community following discharge, there is no population level research that outlines which health care services young and mid-life individuals with complex care needs use following discharge, particularly within the first 7 days. Therefore our study has two broad objectives: to clarify the characteristics of young and mid-life adults discharged from tertiary chronic care and describe their use of health services in the year following hospital discharge; and to examine the association between timely community follow-up and subsequent health outcomes.

## Methods

### Setting and design

This retrospective cohort study was conducted using population-based health administrative databases from the largest province in Canada. Ontario is home to over 13 million people, the vast majority of whom are covered through a universal, publically-funded health insurance program that includes physician services, inpatient care, some home care, and long-term care [23]. Young and mid-life adults aged 18 to 64 years and discharged alive from tertiary chronic care in Ontario, Canada between April 1, 2005 and March 31, 2006 were included in the analyses. The first discharge within this time period was identified as the index date.

### Data sources

The base cohort was derived from the Continuing Care Reporting System (CCRS) [24], which include individual health and functional assessments obtained from the Resident Assessment Instrument Minimum Dataset (RAI-MDS 2.0). The RAI-MDS 2.0 instrument has been widely used to guide care planning and assess quality of care within chronic and long-term care settings [25], and has been internationally tested for reliability and validity within tertiary chronic care, long-term care and home care populations, among others [26–29]. Additional Institute for Clinical Evaluative Sciences (ICES) data holdings were linked to this cohort to provide additional demographic information and to track patterns of health service use by individuals over time. The Registered Persons Database provides demographic information for all Ontarians eligible for public health insurance including date of birth, sex, postal code, and date of death, if applicable. Postal codes were linked to the 2006 Canadian Census to obtain quintiles of neighborhood income level which account for household and community size. Geographic location of residence was identified using the Rurality Index of Ontario. Scores for this index are classified as follows: <10 is considered major urban, 10–39 is considered urban, and a score of ≥40 is considered rural. [30] The Ontario Health Insurance Plan database contains information on inpatient and outpatient physician services. The Canadian Institute for Health Information Discharge Abstract Database provides information on all hospitalizations in Ontario. The National Ambulatory Care Reporting System database records detailed information on all visits to hospital emergency departments. The National Rehabilitation Reporting System contains information on inpatient rehabilitation facilities and programs. The Home Care Database includes information on provincially funded home care services. Provincially funded home care services include nursing, physiotherapy, occupational therapy, speech language pathology, social work, dietetic services, personal support and homemaking (to support activities of daily living). All Ontarians over the age of 18 years of age have access to professional home care services, pending necessity, following an assessment. Despite this practice, not all individuals who need home care receive these services. The Client Profile Database provides information on long-term care home applications and placements. The ICES Physician Database contains data on characteristics of all physicians in Ontario. Access to the linked administrative data was granted through our appointment at ICES (SB). All datasets were linked using unique encoded identifiers and analyzed within ICES according to strict privacy protocols. Approval to complete this study was granted by the Sunnybrook Health Sciences Centre Research Ethics Board. The study was carried out in accordance with the STROBE guidelines.

### Measures

#### Baseline characteristics & functional status

The CCRS assessment closest to the date of discharge (median 30 days, interquartile range (IQR) 8–81) was used to identify clinical and functional RAI-MDS 2.0 assessment information. Socio-demographic characteristics were identified, including age, sex, living status and primary language. RAI-MDS 2.0 can be used to derive several tools to monitor clinical prognosis and outcomes, including the Activities of Daily Living (ADL) Hierarchy Scale [31], the Changes in Health, End-Stage Disease, Signs, and Symptoms (CHESS) Scale [32], the Cognitive Performance Scale (CPS) [33–35], Depression Rating Scale (DRS) [36] the Index of Social Engagement (ISE) [37] and the Aggressive Behaviour Scale (ABS) [38]. All of these data are routinely collected on our population of study and have been validated internationally [29, 39], although predominantly among older adults [29, 39, 40]. The RAI-MDS 2.0 also include a list of active diagnoses present during the assessment and these conditions were considered individually as well as grouped (0, 1, 2, 3+ concurrent conditions). Resource utilization groups (RUG-III 44-group) were used to categorize individuals into a clinical hierarchy based on their resource intensity, from the most resource intensive (special rehabilitation) to the least (reduced physical function) [41].

### Main exposure

#### Community follow-up within 7 days following discharge

Community follow-up within 7 days of discharge was defined as a home care visit, primary care physician visit, both, or neither using mutually exclusive categories. Seven days was chosen for follow-up as it is believed to represent a suitable time window to optimize outcomes including preventing hospital readmissions [18, 42]. Home care visits included skilled nursing, respiratory services, nutrition, physiotherapy, occupational therapy, speech and language, social work, psychology, personal and home care services, placement services, and respite care. Primary care physician visits were identified as consultations and visits to a family physician, defined according to physician specialty, which took place in any of the following outpatient settings, notably, physician office, by phone, at home, or in designated convalescent or chronic care settings.

### Main outcomes

#### Survival following discharge

All-cause mortality rates were identified at 1- and 5- years following discharge.

#### Health system use in the year following discharge

Rates of health service utilization were examined in the year following discharge and included acute care hospital admissions, emergency department visits, admissions to rehabilitation and continuing care facilities, primary care and specialist physician visits, professional home care service visits, as well as long-term care applications and placement. One year was chosen for follow-up given the high mortality rate in this complex, young-to-midlife population and concerns over survival bias.

### Statistical analysis

We compared descriptive differences in demographic, health status, functional characteristics and health system use across type of community-based follow-up (home care, primary care physician visit, both, or neither), using a one-way ANOVA to compare means, the Wilcoxon Rank Sum test to compare medians and chi-square tests for proportions. Five-year survival was examined using Kaplan-Meier survival curves, which were stratified by type of community-based follow-up within 7 days of discharge. Multivariate Cox proportional hazard models [43] were used to compare time to first acute care hospitalization or emergency department visit across types of community follow-up within 1 year of discharge. For each model, individuals were censored at death and first occurrence of complex continuing care and/or rehabilitation admission. For the acute care hospitalization model, first occurrence of an emergency department visit was also included as a censoring variable; for the ED model, first occurrence of an acute care hospitalization was also included as a censoring variable. Adjusted models included age, sex, RUG-III category, number of chronic conditions, and type of conditions as covariates. Analyses were performed with SAS version 9.3 (SAS Institute Inc., Cary, North Carolina). Results were considered significant if the P value was <0.05 (two-tailed). To account for potential bias, explicit inclusion and exclusion criteria for the cohort were pre-specified as part of the analytical plan. Multivariate models were used to account for differences in the distribution of baseline variables across groups.

## Results

### Overall baseline demographic and functional characteristics

Table 1 highlights the complexity of this young and mid-life cohort at discharge from tertiary chronic care. Over one half (54 %) reported 3 or more concurrent chronic conditions; 47 % had a neurological condition; 36 % had a heart condition; and 23 % had a musculoskeletal condition. Half of the cohort (48 %) fell into the Special Rehabilitation category of RUG-III resource utilization intensity. People in this group require some combination of speech, occupational or physiotherapy and restorative nursing care. Approximately three quarters (73 %) of the cohort required some level of assistance with activities of daily living; with 36 % having extensive needs or total dependence. Over half (58 %) had some level of health instability (ranging from mild to very high on the CHESS scale). More than one quarter of the cohort had moderate to severe cognitive impairment, and 22 % exhibited aggressive behaviours. Note that RAI-assessment data were missing for approximately 16 % of the overall cohort.

**Table 1:** Baseline demographic and functional characteristics of young to midlife Ontario adults discharged from complex continuing care between April 1st 2005 and March 31st 2006, by type of community follow-up within 7 days of discharge

	Overall	Home Care Visit Only	Primary Care Physician Visit Only	Both	Neither	*p*-value
Young to midlife adults discharged from complex continuing care, N	1,906	393	631	302	580	
Age (years)						
Mean ± SD	52.0 ± 10.2	52.2 ± 10.4	52.0 ± 9.7	52.6 ± 10.0	51.6 ± 10.6	0.549
18–54	933 (49.0 %)	186 (47.3 %)	306 (48.5 %)	147 (48.7 %)	294 (50.7 %)	0.758
55–64	973 (51.0 %)	207 (52.7 %)	325 (51.5 %)	155 (51.3 %)	286 (49.3 %)	
Sex						
Female	919 (48.2 %)	217 (55.2 %)	277 (43.9 %)	158 (52.3 %)	267 (46.0 %)	0.001
Male	987 (51.8 %)	176 (44.8 %)	354 (56.1 %)	144 (47.7 %)	313 (54.0 %)	
Neighbourhood income quintile						
Q1 (lowest)	547 (28.7 %)	122 (31.0 %)	169 (26.8 %)	94 (31.1 %)	162 (27.9 %)	0.122
Q2	396 (20.8 %)	76 (19.3 %)	132 (20.9 %)	52 (17.2 %)	136 (23.4 %)	
Q3	367 (19.3 %)	67 (17.0 %)	127 (20.1 %)	60 (19.9 %)	113 (19.5 %)	
Q4	327 (17.2 %)	69 (17.6 %)	109 (17.3 %)	50 (16.6 %)	99 (17.1 %)	
Q5 (highest)	250 (13.1 %)	55 (14.0 %)	88 (13.9 %)	38 (12.6 %)	69 (11.9 %)	
Rurality Index of Ontario						
Major urban	1,230 (64.5 %)	231 (58.8 %)	430 (68.1 %)	181 (59.9 %)	388 (66.9 %)	0.004
urban	427 (22.4 %)	116 (29.5 %)	121 (19.2 %)	70 (23.2 %)	120 (20.7 %)	
Rural	201 (10.5 %)	41 (10.4 %)	62 (9.8 %)	42 (13.9 %)	56 (9.7 %)	
Living status						
*Not reported*	*117 (6.1 %)*	*14 (3.6 %)*	*53 (8.4 %)*	*14 (4.6 %)*	*36 (6.2 %)*	0.041
Others	1,331 (69.8 %)	285 (72.5 %)	438 (69.4 %)	207 (68.5 %)	401 (69.1 %)	
Reported living alone	458 (24.0 %)	94 (23.9 %)	140 (22.2 %)	81 (26.8 %)	143 (24.7 %)	
Primary language spoken at home						
Other	151 (7.9 %)	18 (4.6 %)	57 (9.0 %)	22 (7.3 %)	54 (9.3 %)	0.032
English	1,755 (92.1 %)	375 (95.4 %)	574 (91.0 %)	280 (92.7 %)	526 (90.7 %)	
Length of stay in Complex Continuing Care						
Mean ± SD	214.2 ± 797.5	72.9 ± 285.5	376.9 ± 1,131.1	78.3 ± 276.1	203.6 ± 744.0	<.001
Median (IQR)	36 (15–98)	28 (13–57)	59 (20–199)	26 (13–58)	38 (16–96)	<.001
< 30 days	851 (44.6 %)	207 (52.7 %)	220 (34.9 %)	167 (55.3 %)	257 (44.3 %)	<.001
30 to 90 days	542 (28.4 %)	126 (32.1 %)	160 (25.4 %)	86 (28.5 %)	170 (29.3 %)	
> 90 days	513 (27.0 %)	60 (15.2 %)	251 (39.7 %)	49 (16.2 %)	153 (26.4 %)	
Home care referral prior to discharge						
No	1,112 (58.3 %)	106 (27.0 %)	427 (67.7 %)	84 (27.8 %)	495 (85.3 %)	<.001
Yes	794 (41.7 %)	287 (73.0 %)	204 (32.3 %)	218 (72.2 %)	85 (14.7 %)	
Health conditions/diagnoses						
Endocrine/Metabolic/Nutritional	527 (27.6 %)	118 (30.0 %)	166 (26.3 %)	93 (30.8 %)	150 (25.9 %)	0.251
Heart/Circulation	689 (36.1 %)	155 (39.4 %)	217 (34.4 %)	119 (39.4 %)	198 (34.1 %)	0.165
Musculoskeletal	428 (22.5 %)	116 (29.5 %)	123 (19.5 %)	74 (24.5 %)	115 (19.8 %)	<.001
Neurological	889 (46.6 %)	153 (38.9 %)	356 (56.4 %)	120 (39.7 %)	260 (44.8 %)	<.001
Psychiatric/Mood	534 (28.0 %)	92 (23.4 %)	208 (33.0 %)	83 (27.5 %)	151 (26.0 %)	0.005
Pulmonary	212 (11.1 %)	44 (11.2 %)	55 (8.7 %)	44 (14.6 %)	69 (11.9 %)	0.053
Sensory	83 (4.4 %)	13 (3.3 %)	38 (6.0 %)	8 (2.6 %)	24 (4.1 %)	0.06
Other	817 (42.9 %)	185 (47.1 %)	257 (40.7 %)	139 (46.0 %)	236 (40.7 %)	0.095
Number of concurrent conditions						
0	51 (2.7 %)	13 (3.3 %)	16 (2.5 %)	9 (3.0 %)	13 (2.2 %)	0.242
1	241 (12.6 %)	43 (10.9 %)	79 (12.5 %)	33 (10.9 %)	86 (14.8 %)	
2	272 (14.3 %)	60 (15.3 %)	93 (14.7 %)	35 (11.6 %)	84 (14.5 %)	
3+	1,034 (54.2 %)	212 (53.9 %)	358 (56.7 %)	174 (57.6 %)	290 (50.0 %)	
missing	308 (16.2 %)	65 (16.5 %)	85 (13.5 %)	51 (16.9 %)	107 (18.4 %)	
Resource Utilization Group (RUG-III class)						
Special Rehabilitation	917 (48.1 %)	199 (50.6 %)	296 (46.9 %)	160 (53.0 %)	262 (45.2 %)	0.001
Extensive Services	91 (4.8 %)	15 (3.8 %)	25 (4.0 %)	9 (3.0 %)	42 (7.2 %)	
Special Care	232 (12.2 %)	47 (12.0 %)	84 (13.3 %)	44 (14.6 %)	57 (9.8 %)	
Clinically Complex	253 (13.3 %)	49 (12.5 %)	95 (15.1 %)	29 (9.6 %)	80 (13.8 %)	
Functional Impairment	73 (3.8 %)	10 (2.5 %)	35 (5.5 %)	7 (2.3 %)	21 (3.6 %)	
*missing*	340 (17.8 %)	73 (18.6 %)	96 (15.2 %)	53 (17.5 %)	118 (20.3 %)	
Activity of Daily Living Self-Performance Hierarchy						
0	203 (10.7 %)	44 (11.2 %)	53 (8.4 %)	35 (11.6 %)	71 (12.2 %)	<.001
1–3	710 (37.3 %)	160 (40.7 %)	220 (34.9 %)	127 (42.1 %)	203 (35.0 %)	
4–6	685 (35.9 %)	124 (31.6 %)	273 (43.3 %)	89 (29.5 %)	199 (34.3 %)	
*missing*	308 (16.2 %)	65 (16.5 %)	85 (13.5 %)	51 (16.9 %)	107 (18.4 %)	
Cog4nitive Performance Scale						
0–2	1,087 (57.0 %)	266 (67.7 %)	301 (47.7 %)	200 (66.2 %)	320 (55.2 %)	<.001
3+	511 (26.8 %)	62 (15.8 %)	245 (38.8 %)	51 (16.9 %)	153 (26.4 %)	
*missing*	308 (16.2 %)	65 (16.5 %)	85 (13.5 %)	51 (16.9 %)	107 (18.4 %)	
Changes in Health, End-Stage Disease, Signs, and Symptoms Scale						
0	488 (25.6 %)	105 (26.7 %)	176 (27.9 %)	66 (21.9 %)	141 (24.3 %)	0.276
1–3	1,024 (53.7 %)	209 (53.2 %)	344 (54.5 %)	169 (56.0 %)	302 (52.1 %)	
4+	86 (4.5 %)	14 (3.6 %)	26 (4.1 %)	16 (5.3 %)	30 (5.2 %)	
*missing*	308 (16.2 %)	65 (16.5 %)	85 (13.5 %)	51 (16.9 %)	107 (18.4 %)	
Depression Rating Scale						
0–2	1,219 (64.0 %)	258 (65.6 %)	411 (65.1 %)	195 (64.6 %)	355 (61.2 %)	0.329
3+	364 (19.1 %)	68 (17.3 %)	129 (20.4 %)	54 (17.9 %)	113 (19.5 %)	
*missing*	323 (16.9 %)	67 (17.0 %)	91 (14.4 %)	53 (17.5 %)	112 (19.3 %)	
Index of Social Engagement						
0–2	644 (33.8 %)	108 (27.5 %)	265 (42.0 %)	85 (28.1 %)	186 (32.1 %)	<.001
3+	954 (50.1 %)	220 (56.0 %)	281 (44.5 %)	166 (55.0 %)	287 (49.5 %)	
*missing*	308 (16.2 %)	65 (16.5 %)	85 (13.5 %)	51 (16.9 %)	107 (18.4 %)	
Aggressive Behaviour Scale						
0	1,161 (60.9 %)	261 (66.4 %)	373 (59.1 %)	188 (62.3 %)	339 (58.4 %)	0.005
1+	422 (22.1 %)	65 (16.5 %)	167 (26.5 %)	61 (20.2 %)	129 (22.2 %)	
*missing*	323 (16.9 %)	67 (17.0 %)	91 (14.4 %)	53 (17.5 %)	112 (19.3 %)	

*SD* standard deviation, *IQR* interquartile range

### Patterns of community follow-up

As noted in Table 2, most individuals received some form of follow-up care within 7 days of discharge; 21 % received home care only, 33 % had a primary care physician visit only, 16 % received both, and close to one third (30 %) received neither. In general, demographic and functional characteristics did not differ across these community follow-up groups. Those who received home care (only or with a primary care physician visit) were more likely to be female, less likely to live in major urban areas, and had much shorter median lengths of stay than those with primary care physician visits only or neither. Individuals who received a home care visit within 7 days of discharge were also more likely to have a musculoskeletal condition, less likely to have a neurological condition and less likely to have high functional impairment than those who received a primary care physician visit only or neither.

**Table 2:** Mortality and health system use among young to midlife Ontario adults discharged from complex continuing care between April 1st 2005 and March 31st 2006, by type of community follow-up within 7 days of discharge

	Overall	Home Care Visit Only	Primary Care Physician Visit Only	Both	Neither	*p*-value
Young to midlife adults discharged from complex continuing care, N	1,906	393	631	302	580	
Number of deaths, n (%)						
Within 7 days	52 (2.7 %)	0 (0.0 %)	15 (2.4 %)	12 (4.0 %)	25 (4.3 %)	<.001
Within 1 year	337 (17.7 %)	61 (15.5 %)	88 (13.9 %)	76 (25.2 %)	112 (19.3 %)	<.001
Within 5 years	787 (41.3 %)	149 (37.9 %)	269 (42.6 %)	142 (47.0 %)	227 (39.1 %)	0.057
Acute Care & Psychiatric Hospital Admissions						
Any admission, n (%)	981 (51.5 %)	197 (50.1 %)	302 (47.9 %)	128 (42.4 %)	354 (61.0 %)	<.001
Mean ± SD	1.9 ± 1.6	1.9 ± 1.4	1.7 ± 1.2	2.2 ± 1.9	1.9 ± 1.9	0.053
Median (IQR)	1 (1–2)	1 (1–2)	1 (1–2)	1 (1–3)	1 (1–2)	0.025
Acute length of stay						
Mean ± SD	19.8 ± 84.5	13.3 ± 30.8	23.1 ± 104.9	17.8 ± 108.5	21.4 ± 75.1	0.613
Median (IQR)	7 (4–13)	7 (4–12)	6 (4–12)	6 (3–10)	9 (4–15)	<.001
Acute Care Alternate Level of Care (ALC) Admissions						
Any ALC, n (%)	196 (10.3 %)	54 (13.7 %)	42 (6.7 %)	23 (7.6 %)	77 (13.3 %)	<.001
ALC length of stay						
Mean ± SD	23.0 ± 40.6	17.2 ± 23.6	30.6 ± 62.8	8.2 ± 7.7	27.3 ± 39.4	0.089
Median (IQR)	10 (4–24)	11 (5–19)	8 (6–19)	6 (4–10)	15 (5–33)	0.049
Any Same-day Surgery Visits, n (%)	309 (16.2 %)	67 (17.0 %)	96 (15.2 %)	47 (15.6 %)	99 (17.1 %)	0.784
Mean ± SD	1.6 ± 1.9	1.8 ± 2.3	1.5 ± 1.3	1.5 ± 1.1	1.8 ± 2.4	0.664
Median (IQR)	1 (1–2)	1 (1–2)	1 (1–2)	1 (1–2)	1 (1–2)	0.296
Emergency Department Visits						
Any visit, n (%)	1,204 (63.2 %)	265 (67.4 %)	374 (59.3 %)	191 (63.2 %)	374 (64.5 %)	0.055
Mean ± SD	10.6 ± 29.4	8.9 ± 24.3	10.5 ± 30.5	11.0 ± 28.5	11.6 ± 31.8	0.713
Median (IQR)	2 (1–5)	3 (1–6)	2 (1–4)	3 (1–7)	2 (1–5)	0.006
Inpatient Rehabilitation Admissions						
Any admission, n (%)	263 (13.8 %)	25 (6.4 %)	72 (11.4 %)	15 (5.0 %)	151 (26.0 %)	<.001
Mean ± SD	1.1 ± 0.3	1.1 ± 0.4	1.2 ± 0.4	1.1 ± 0.4	1.1 ± 0.2	0.103
Median (IQR)	1 (1–1)	1 (1–1)	1 (1–1)	1 (1–1)	1 (1–1)	0.151
Complex Continuing Care Admissions						
Any admission, n (%)	389 (20.4 %)	55 (14.0 %)	160 (25.4 %)	32 (10.6 %)	142 (24.5 %)	<.001
Mean ± SD	7.0 ± 7.6	3.7 ± 5.5	8.7 ± 8.2	2.6 ± 2.8	7.3 ± 7.4	<.001
Median (IQR)	4 (2–9)	2 (1–3)	6 (3–11)	1 (1–3)	5 (2–10)	<.001
Primary Care Physician Visits						
Any visit, n (%)	1,764 (92.5 %)	356 (90.6 %)	631 (100.0 %)	302 (100.0 %)	475 (81.9 %)	<.001
Mean ± SD	21.0 ± 27.1	9.1 ± 9.6	32.0 ± 32.9	14.0 ± 13.4	19.6 ± 28.3	<.001
Median (IQR)	11 (5–24)	6 (3–12)	20 (9–44)	11 (5–18)	8 (4–19)	<.001
Specialist Visits						
Any visit, n (%)	1,425 (74.8 %)	307 (78.1 %)	447 (70.8 %)	230 (76.2 %)	441 (76.0 %)	0.04
Mean ± SD	6.9 ± 9.8	6.8 ± 7.0	5.9 ± 8.6	7.0 ± 7.3	7.8 ± 13.1	0.034
Median (IQR)	4 (2–8)	5 (2–9)	4 (2–7)	5 (2–10)	4 (2–9)	0.003
Home Care Service Use						
Any home care service use, n (%)	626 (32.8 %)	252 (64.1 %)	89 (14.1 %)	176 (58.3 %)	109 (18.8 %)	<.001
Mean ± SD	19.0 ± 19.7	23.7 ± 22.2	13.5 ± 17.3	18.9 ± 17.7	12.6 ± 15.2	<.001
Median (IQR)	12 (6–26)	18 (9–31)	7 (3–18)	14 (6–25)	7 (4–14)	<.001
Long-Term Care Use						
Any long-term care applications, n (%)	138 (7.2 %)	36 (9.2 %)	42 (6.7 %)	16 (5.3 %)	44 (7.6 %)	0.233
Any long-term care placements, n(%)	300 (15.7 %)	21 (5.3 %)	196 (31.1 %)	14 (4.6 %)	69 (11.9 %)	<.001

*SD* standard deviation, *IQR* interquartile range

### Survival across follow-up groups

**Fig. 1:**
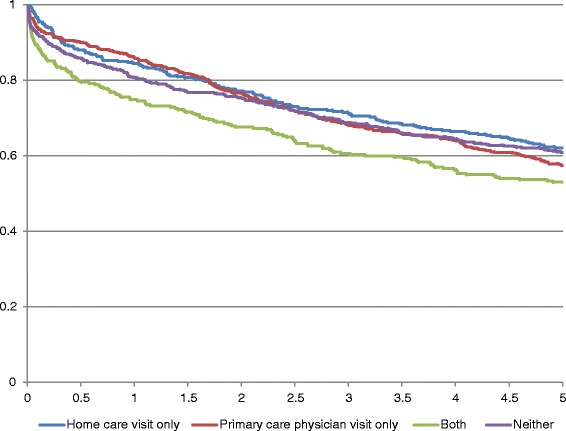
Kaplan-Meier 5-year survival curves for young to midlife Ontario adults discharged from complex continuing care between April 1st 2005 and March 31st 2006, by type of community follow-up within 7 days of discharge

Approximately 18 % of the cohort died within the year following discharge and 41 % had died after 5 years (Table 2). Figure 1 illustrates that survival differed across community follow-up groups at 1 year. Mortality was highest among individuals who received both home care and primary care visits within 7 days (25 %). Neoplasms (45 %) (i.e., cancer-related) followed by diseases of the circulatory system (13 %), were the most common cause of death within 1 year, overall and across follow-up groups (data not shown).

### Health system utilization across follow-up groups

Over half (52 %) of individuals had at least one acute care admission in the year following discharge (Table 2). Across community follow-up groups, those with neither home care nor primary care physician follow-up within 7 days had the highest proportion of hospitalizations.

Over half of the cohort (63 %) had at least one ED visit (median number of visits 2, IQR 1–5) in the year following discharge; and 20 % were re-admitted to tertiary chronic care (median number of readmissions = 4, IQR = 2–9). Across the community follow-up groups those who received home care only had the highest proportion of ED visits. The highest proportion of complex continuing care readmissions were seen in the physician follow-up group.

The majority of the cohort (93 %) visited a primary care physician within the first year (median number of visits 11, IQR 5–24) and 75 % saw a specialist (median number of visits 4, IQR 2–8).

Across the follow-up groups, those in the physician only group as well as the physician and home care follow-up group had the highest proportion of physician visits (meaning, beyond the 7 days follow-up, they were most likely, compared to the other groups to have physician visits over the course of the year). Those in the home care only follow-up group had the highest proportion of specialist visits.

One third of the cohort (33 %) (median number of visits 12, IQR 6–26) used professional home care services in the year following discharge. Close to one-fifth (16 %) of the cohort was placed into a long-term care facility within the first year. Across the community follow-up groups, those in the home care only category had the highest proportion of home care visits (meaning, they had more than just short-term home care and continued to use home care over the course of the year). Those in the primary care physician visit only follow-up group had the highest proportion of long-term care placements.

### Effect of community follow-up on health service use 1-year following discharge

After adjusting for individual demographic and clinical characteristics (Table 3), results indicate the time to acute care hospitalization in the year following discharge was significantly longer among those who received both a home care and a primary care physician follow-up visit compared to those who did not receive any form of community follow-up (HR 0.38, 95%CI 0.26–0.53). This finding held among the other community follow-up groups as well relative to no follow-up: primary care physician visit only (HR 0.64, 95%CI 0.50–0.80); and home care only (HR 0.40, 95%CI 0.29–0.54). No significant associations were found between community follow-up and time to ED visit within 1 year.

**Table 3:** Adjusted risk of acute care hospitalization and emergency department use within one-year of discharge from CCC, by type of community follow-up within 7 days of discharge

	Inpatient hospitalization		ED visit	
	HR (95 % CI)	*p*-value	HR (95 % CI)	*p*-value
Home Care Visit Only	0.40 (0.29, 0.54)	<.0001	0.89 (0.72, 1.10)	0.28
Primary Care Physician Visit	0.64 (0.50, 0.80)	<.001	0.82 (0.68, 1.00)	0.05
Both	0.38 (0.26, 0.53)	<.0001	0.95 (0.77, 1.19)	0.68
Neither	1.0		1.0	

Cox proportional hazards model adjusted for age, sex, income, rurality, RUG-III group, number of conditions, type of conditions

*Note*: Due to missing information on functional status

## Discussion

This study focused on a young and mid-life adult population with complex care needs discharged from high intensity tertiary chronic care (referred to as Complex Continuing Care in Ontario). First, we found that these individuals were characterized by multi-morbidity, functional deficits, lower socio-economic status and poor survival. Second, we discovered an overall high use of hospital and physician care within the year following discharge with much lower uptake of professional home care support. Third, we found that follow-up care in the community within 7 days (either home care or primary care physician visit) may reduce subsequent acute care hospital admissions but not emergency department use and mortality.

This cohort reflected a high prevalence of multi-morbidity, compounded by low socio-economic status, social isolation, cognitive impairment and/or behavioral disorders for some. While we did not examine the clustering of physical, mental and social conditions within individuals, some degree of overlap is presumed. The characteristics of our cohort are similar to those presented in other studies. For instance, complexity of care needs, particularly multi-morbidity at a young age, is not uncommon. Barnett et al. [44] have noted that the prevalence of multi-morbidity increases with age, but the absolute number of people with multi- morbidity is highest among the under 65 population. Further, Barnett et al. found that being in a lower socio-economic stratum (as found in half of our population) may be associated with earlier onset of morbidities (as much as 10–15 years) before others who are from the highest income quintiles. Thus, what we see in our population as well as similar populations internationally is a constellation of factors, both health and social in nature, that may coalesce to create ongoing reliance on healthcare [44–46], even at a young age.

Overall, the young and mid-life adults in our cohort used a substantial number of health care services following discharge from tertiary chronic care, particularly hospital, emergency department and primary care practitioner and specialist visits.

The extensive use of hospital and emergency department services, even in the groups with timely community follow-up, may be indicative of ongoing health instability following tertiary chronic care discharge. A high level of contact with primary care physicians and specialists is not surprising given the levels of functional impairment exhibited in our population. As noted in previous research, being connected with a consistent care provider/ care team in the community (sometimes referred to as a “medical home”) at discharge can reduce unnecessary repeat use of hospital services, reduce mortality and optimize the care and functioning of people, particularly with complex care needs [22]. Future studies should examine the extent to which complex young and mid-life adults are deliberately connected to a consistent provider in the community, for ongoing chronic disease management and the impact that this has on overall utilization of care.

For a high-need population with significant complexity, use of home care services in the year following discharge was surprisingly low in our cohort. Specific sub-groups within our cohort, including those with significant ADL needs and neurological conditions were less likely to use home care calling into question whether services are accessible to these populations in the community. While home care may have the potential to offset use of other more expensive services, such as long-term care, only 42 % of the cohort had a home care referral at discharge and 33 % used home care within 1 year. Among those referred, only 63 % received a service within 7 days (referred to as post-acute or short-term home care) and approximately two thirds continued to receive home care services over the course of the year.

Limited use of professional home care might highlight a lack of availability, resistance to uptake, or lack of perceived appropriateness of services; findings which have been seen in studies involving older adults with multi-morbidities [47–49]. Furthermore, whether or not use of hospital and emergency services was used in the absence of timely access to care in the home or by a primary care provider (“upward substitution”) is unclear and requires further research. Such trends of upward substitution to hospital, emergency rooms and long-term care have been found in previous research involving older adult populations when unable to access timely home and community care [49–51].

When we examined timely follow-up, almost three quarters of the cohort visited a primary care physician and/or had a home care visit within the 7 days of discharge. Timely follow-up care by a physician or services in the home has been shown to mitigate hospital re-admission in previous research [18–21]. In this study, our data suggest that community follow-up by a physician, home care or both were protective against acute care admission within 1 year. These findings held after controlling for individual personal and illness characteristics (see Table 3 for the adjusted hazard ratios).

Immediate follow-up care did not appear to be protective against emergency services use and mortality. In fact, those who received both types of follow-up care (physician and home care) had the greatest likelihood of mortality at both 1 and 5 years following hospital discharge. It is interesting to note that mortality was highest among those who were (for some period) in the home care and physician care follow-up group. This is in contrast to a study by Fidahussein [5] where post-discharge follow-up for COPD patients resulted in lower mortality, albeit no significant reductions in emergency department and hospital readmissions.

In understanding the high mortality rate among those initially referred to both types of follow-up care, two factors could be at play. First, selection bias may have played a role in our study if the most complex patients were referred to and received the most immediate follow-up care. In this case, earlier mortality may have been inevitable due to their complex conditions and potentially poor prognosis. On the other hand, while the cohort had numerous contacts with the health care system it is not clear if care was well-managed, properly coordinated or appropriate (in terms of type and volume of services provided). Previous studies have suggested that more access to health care, does not necessarily equate to better outcomes [52, 53]. As health care utilization increases so too does the risk of adverse events, including poorly executed transitions and medication reconciliation problems [54]. Furthermore, a lack of clinical practice guidelines (CPGs) and the application of single disease guidelines to multi-morbid populations (such as ours) can lead to adverse outcomes [55–57]. These assertions extend beyond our data findings, but represent an important area of further inquiry.

### Limitations

There are a number of limitations to this study. The Scales derived from the MDS suite of instruments (ADL Hierarchy Scale, etc.) have been validated primarily among older adults with further research required for the young and mid-life adult population with complex care needs. However, we emphasize that factors such as multimorbidity and functional status have been shown to be stronger predictors of health care utilization than age [58].

Furthermore, population-based administrative datasets do not provide the full scope of data required to garner a comprehensive understanding of the motivations driving health service use or the context and coordination of service delivery across sectors. However, this study does provide a broad overview of patterns of system use by clinically complex young and mid-life adults. The authors did not have access to non-professional home support data, which would include care provided by family caregivers, volunteers, not-for-profit community agencies and private providers. Support from these sources, particularly informal caregivers likely played a role in meeting the needs of this population. Further, the way in which these informal supports may have substituted or complemented formal supports is unknown. Although ongoing reforms in health services, particularly physician care [59, 60] have taken place since the timeframe of the analysis recent studies continue to show major gaps in care, poor care continuity [61], issues regarding access to timely care and lack of physician comfort in dealing with this population [62]. Given these realities we are uncertain whether more recent data would have changed our findings.

The illness characteristics of people in the study (including functional and cognitive status, diagnoses, mental health characteristics and RUG grouping) were captured as close as possible to hospital discharge using data from the Resident Assessment Instrument 2.0. In tertiary chronic care in Ontario, patient data from this source are updated every 3 months; and over 2 months had elapsed between the collection of these data and the actual date of discharge for a proportion of the cohort. However, given the persistent use of health services over time, the authors assume that the needs of the cohort under study remained relatively high. Finally, in our dataset we were unable to assess the quality or type of intervention that may have been included as part of a follow-up package of service by a physician or home care provider. Research by Naylor [20] and Coleman [19] demonstrate that coaching from a nurse during care transitions followed by home support was protective against acute care admissions for older adults with complex care needs compared to those who received standard discharge planning and follow-up care. In our study, the availability of high quality post hospital interventions could have altered the outcomes somewhat, particularly related to emergency department visits.

### Conclusions and recommendations

This study adds important baseline data on care use and post-hospital follow-up on a young and mid-life adult population with complex care needs. While the presence of chronic disease and care use is expected (and has been extensively studied) among older adults, young and mid-life adults have received far less attention in the literature. Our study shows that young and mid-life adults who are discharged from tertiary chronic care appear to have ongoing impairments, mortality risk and high health system use. Use of home care services is low relative to hospital, emergency room and physician visits in the year following discharge. Adding to the literature on the importance of post-hospital follow-up our study suggests that immediate connection to home care and/or primary physician care within the first week of discharge may protect against and delay future acute care system use.

We provide a number of recommendations based on these findings for future policy and research. First, while heavy use of care is evident in this population, continuity of care (i.e., the extent to which patients had access to consistent providers over a period of time) is not. Considering that seeing a consistent care provider may mitigate inappropriate use of hospital and emergency services, this needs to be further explored. Second, exploring why utilization of home care was low may lend itself to a different methodological approach such as qualitative interviews where respondents can share reasons of use/non-use and gaps. Also, assessing the availability of non-professional home care supports, including the availability and quality of care from informal care providers (e.g., family) is required to better understand the full scope of supports used by this population as well as how informal caregivers are handing complex care. Finally, understanding why immediate follow-up is not protective against emergency services use and mortality needs to be further explored to see if these outcomes can be avoided (emergency services use) or prolonged (mortality) through greater quality of care and follow-up.

While our study aims to provide important baseline data on service use and the impact of follow-up care for young and midlife adults following discharge from tertiary chronic care, future research is required to determine the extent to which post-discharge options are appropriately tailored to the needs of the young and mid-life chronic care population as well as potential care gaps. In doing so, a more in-depth understanding can be achieved on the characteristics, needs and utilization patterns of this population to guide quality improvement in the health care system for this population.
